# Silica nanochannels boosting Ru(bpy)_3_^2+^-mediated electrochemical sensor for the detection of guanine in beer and pharmaceutical samples

**DOI:** 10.3389/fnut.2022.987442

**Published:** 2022-08-30

**Authors:** Luoxing Yang, Tongtong Zhang, Huaxu Zhou, Fei Yan, Yan Liu

**Affiliations:** ^1^Department of Chemistry, Key Laboratory of Surface and Interface Science of Polymer Materials of Zhejiang Province, Zhejiang Sci-Tech University, Hangzhou, China; ^2^Department of Hepatobiliary and Pancreatic Surgery, The Center for Integrated Oncology and Precision Medicine, Affiliated Hangzhou First People’s Hospital, Zhejiang University School of Medicine, Hangzhou, China; ^3^Department of Breast, Bone and Soft Tissue Oncology, Laboratory of Breast Cancer Diagnosis and Treatment Research of Guangxi Department of Education, Guangxi Medical University Cancer Hospital, Affiliated Tumor Hospital of Guangxi Medical University, Nanning, China

**Keywords:** vertically ordered mesoporous silica film, Ru(bpy)_3_**^2+^**, electrochemical detection, guanine, beer, pharmaceutical

## Abstract

Vertically ordered mesoporous silica film (VMSF) with uniform mesoporous channels perpendicular to electrode substrate has a wide range of applications in direct electroanalysis of complex samples. However, the detection of nucleic acid bases is difficult to realize at the commonly used VMSF-modified indium tin oxide (VMSF/ITO) electrode due to the high overpotentials of underlying ITO for many small organic molecules. In this work, we demonstrated an electrochemical method for the sensitive detection of guanine (G) by integration of VMSF/ITO and tris(2,2′-bipyridine) ruthenium (II) [Ru(bpy)_3_^2+^] redox mediator. Ru(bpy)_3_^2+^ electrostatically accumulated by VMSF is able to act as an electron shuttle between G and underlying ITO surface, showing electrocatalytic oxidation of G and enabling the quantitative determination of G with a limit of detection (LOD) of 0.058 μM and a limit of quantitation (LOQ) of 0.2 μM. Electrochemical detection performance for G could be regulated by changing the pH of the supporting electrolyte and the content of Ru(bpy)_3_^2+^, achieving a wide dynamic linear range from 0.2 to 10 μM (*R*^2^ = 0.999), 2 to 100 μM (*R*^2^ = 0.999), and 10 to 500 μM (*R*^2^ = 0.998). Furthermore, owing to the good anti-fouling and anti-interference ability of VMSF, this simply sensing strategy can be applied to the direct and rapid detection of G in beer samples, and the detection of ganciclovir (G analog) content in ganciclovir eye drops.

## Introduction

Guanine (G), a vital component of cells, plays a crucial role in energy transduction, metabolic cofactors, and cell signaling (1). G is also an important purine base existed in biological molecules, such as deoxyribonucleic acid (DNA) and ribonucleic acid (RNA) (2). It has been reported that G is easily oxidized by free radicals or certain oxidants. Therefore, the variation in the concentration of G is able to reflect the degree of DNA oxidative damage and can be regarded as an important parameter in diagnosing various diseases, such as epilepsy, cancer, and acquired immune deficiency syndrome (3–5). In addition, the abundance of G in food and beverage (e.g., seafood and beer) is well established, and it will be eventually metabolized to uric acid in the human body, probably leading to the gout. Therefore, the detection of G level is of extremely important in clinical diagnosis. At present, there are many techniques for the detection of G, such as calorimetry (6), high-performance liquid chromatography (HPLC) (4), laser-induced fluorescence (7), isotope dilution mass spectrometry (8), and capillary electrophoresis (9). Among them, HPLC is the most common detection method for G (10), but this detection strategy is expensive, time-consuming, and requires professional operator and expensive instruments. Compared with HPLC, electrochemical methods have the advantages of fast detection, high sensitivity, and low cost. Considering the high oxidation potential of G on electrode, different nanomaterials are modified on the electrode to achieve the detection of G (11–13). These methods require modification of complex nanomaterials, increasing the difficulty of sensor construction. However, direct electrochemical reactivity of G on the bare working electrodes is poor, displaying slow electron transfer and ultimately resulting in the low selectivity and sensitivity (14). Therefore, designing new electrochemical sensing strategies for G detection is highly desirable.

Since Johnston et al. (15, 16) discovered that oxidized tris(2,2′-bipyridine) ruthenium (III) [Ru(bpy)_3_^3+^] could mediate the electrochemical oxidation of G residues in DNA, many label-free electrochemical sensors based on this characteristic of G have been developed. For example, Kim et al. (17). reported a novel “ON–OFF” electrochemical method for determining adenosine (A). The detection relies on the conformational difference between a random aptamer and the tertiary structure of the aptamer-A complex. The enhanced electrochemical oxidation of Ru(bpy)_3_^2+^ by a G-rich and A-sensitive aptamer could be decayed by the formation of aptamer-A complex, leading to the decreased signals associated with the reduced accessibility of Ru(bpy)_3_^2+^ mediator to the G. Moreover, Dang et al. (18). have designed a switch for the electrochemiluminescence (ECL) detection of K^+^ based on the G-rich DNA aptamer and chitosan/Ru(bpy)_3_^2+^/silica (CRuS) nanoparticle (NP)-modified glass carbon electrode (GCE). In the presence of G-rich DNA aptamer with unfold state, enhancement of ECL signal was observed at the CRuS NPs/GCE. Upon addition of K^+^, G-quadruplex structure was formed, which greatly affected the ECL enhancing effect of G to the ECL reaction of Ru(bpy)_3_^2+^. On the basis of this sensing mechanism, label-free and sensitive ECL detection of K^+^ in colorectal cancer cells was realized. As the electrochemical activity of the working electrode for Ru(bpy)_3_^2+^ is closely related to the detection performance, construction of electrochemical sensing interfaces with high sensitivity and low cost is necessary.

Porous materials have aroused increasing attention in the development of various high-performance sensors (19–21), drug delivery systems (22), and electrocatalysts (23). Vertically ordered mesoporous silica film (VMSF) as an attractive electrode modification material has ultrasmall and uniform pore diameter, high porosity, and ordered silica nanochannel perpendicular to the electrode substrate, exhibiting high permeability and excellent molecular selectivity (e.g., size, charge, hydrophobicity, and isomer) and showing a wide range of applications in direct electroanalysis of complex samples (24–26). There are three main methods for the growth of VMSF on solid electrodes, including Stöber-solution (27–29), biphasic stratification (30), and electrochemically assisted self-assembly (EASA) (31–34) methods, of which EASA method can complete the modification in a few seconds and the obtained VMSF has more regular mesopores. Arising from the rich silanol groups (p*K*_*a*_∼2) on the channels, VMSF with negatively charged surface is able to accumulate the positively charged Ru(bpy)_3_^2+^ through electrostatic interaction (35–38), which has displayed satisfactory performance in Ru(bpy)_3_^2+^ luminophore-based ECL analytical systems. However, stable fabrication of VMSF on conventional electrodes [e.g., glassy carbon (39, 40), 3D graphene (41, 42), and gold (43, 44)] requires a special adhesive layer or pretreatment procedure, avoiding modified VMSF from the falling off the substrate electrodes over long-term use. By contrast, indium tin oxide (ITO), a kind of suitable electrode to support VMSF due to their formation of O-Si-O chemical bonds, can greatly improve the long-term stability of the modified electrode, and electrode area can be tuned by cutting the ITO in a desirable size (45). Therefore, direct exploitation of VMSF-modified ITO (VMSF/ITO) electrode in electrochemical analysis has gained superiority. However, high overpotentials of many small organic molecules occur at the underlying ITO electrode, limiting the broadened applications of VMSF/ITO.

In this work, VMSF/ITO electrode was facilely prepared using EASA method and integrated with Ru(bpy)_3_^2+^ redox mediator to electrochemically detect G. Owing to the charge permselectivity of VMSF, Ru(bpy)_3_^2+^ could be electrostatically enriched to the nanochannels of VMSF and adsorbed Ru(bpy)_3_^2+^ served as an electron mediator participates in the electrochemical oxidation of G. The quantitative determination of G was realized by recording the electrocatalytic currents. The effect of the pH of the supporting electrolyte and the content of Ru(bpy)_3_^2+^ on the detection performance were investigated. The practical application of our proposed Ru(bpy)_3_^2+^-mediated VMSF sensor in the accurate and prompt detection of G in beer samples, and the detection of ganciclovir (G analog) content in ganciclovir eye drops were studied with satisfactory results.

## Materials and methods

### Chemicals and materials

Tetraethoxysilane (TEOS), cetyltrimethylammonium bromide (CTAB), potassium ferricyanide (K_3_[Fe(CN)_6_]), sodium phosphate dibasic heptahydrate (Na_2_HPO_4_⋅7H_2_O), sodium phosphate monobasic (Na_2_H_2_PO_4_), glucose (Glu), uric acid (UA), bovine serum albumin (BSA), tris-hydrochloride buffer (Tris–HCl), phosphoric acid (H_3_PO_4_), boric acid (H_3_BO_3_), acetic acid (HAc), sodium acetate trihydrate (NaAc), and potassium hydrogen phthalate (KHP) were bought from Aladdin. Hexaammineruthenium(III) chloride [Ru(NH_3_)_6_Cl_3_] and tris(2,2′-bipyridine)dichlororuthenium(II) hexahydrate [Ru(bpy)_3_Cl_2_⋅6H_2_O, 98%] were ordered from Sigma-Aldrich. Guanine (G), adenine (A), thymine (T), and cytosine (C) were purchased from Macklin. Sodium nitrate (NaNO_3_) was obtained from Wuxi Zhanwang Chemical Reagent. Potassium chloride (KCl) was purchased from Hangzhou Gaojing Fine Chemical Co., Ltd. (Hangzhou, China). Urea was purchased from Tianjin Yongda Chemical Reagent Co., Ltd. (Tianjin, China). Ganciclovir eye drops were purchased from Hubei Yuanda Tiantianming Pharmaceutical Co., Ltd. Beer samples were purchased from the supermarket. All chemicals and reagents of analytical grade were used as received without further purification, and ultrapure water (18.2 MΩ⋅cm) was used to prepare all aqueous solutions throughout this work.

### Measurements and instrumentations

Transmission electron microscopy (TEM) images were obtained on a JEM-2100 microscope (JEOL, Japan) at an acceleration voltage of 200 kV. VMSF was mechanically peeled off from the ITO electrode surface, dispersed into ethanol, and finally dropped onto copper grids, to obtain TEM specimen. Scanning electron microscopy (SEM) images were collected from the SU8100 microscope (Hitachi, Japan) at an acceleration voltage of 10 kV. Cyclic voltammetry (CV) was taken on an Autolab PGSTAT302N electrochemical workstation (Metrohm, Switzerland). A conventional three electrodes system was employed with bare ITO or modified ITO electrode as the working electrode, an Ag/AgCl electrode (saturated with KCl) as the reference electrode, and a platinum electrode as the counter electrode.

### Preparation of the vertically ordered mesoporous silica film/indium tin oxide electrode

**SCHEME 1:**
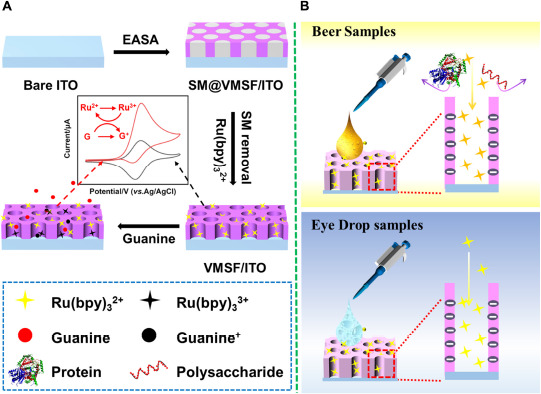
**(A)** The preparation of VMSF/ITO electrode and electrochemical detection of G. **(B)** Electrochemical detection of G in beer samples and ganciclovir in eye drop samples.

Vertically ordered mesoporous silica film/ITO was prepared by using the EASA approach as previously reported in literature (Scheme 1) (31). Briefly, the clean ITO electrode (0.5 cm × 1.0 cm) was immersed into the EASA precursor containing 1.585 g CTAB, 3050 μL TEOS, 20 mL ethanol, and 20 mL NaNO_3_ (pH = 2.6). Then, a constant current of –350 μA was applied to the bare ITO electrode for 10 s using a common three-electrode system, namely bare ITO electrode as the working electrode, platinum sheet as the counter electrode, and Ag/AgCl electrode (saturated with KCl) as the reference electrode. After being rinsed with copious amounts of water, dried under a N_2_ stream, and further aged at 120°C overnight, the resulting electrode with surfactant micelles (SM) inside the nanochannels was named as SM@VMSF/ITO. SM could be removed by immersing the SM@VMSF/ITO into a 0.1 M HCl–ethanol solution under stirring for 5 min to obtain VMSF/ITO.

### Electrochemical detection of guanine

The VMSF/ITO electrode was first immersed into a 0.01 M PBS (pH = 7.0) solution containing redox mediator Ru(bpy)_3_^2+^ of certain concentration (0.1, 1, or 10 μM) and underwent mechanical stirring to reach plateau of redox signals. Then, G with various concentrations was added to the above solution and detected by CV technique. The scan rate was 100 mV/s.

## Results and discussion

### Characterization of the vertically ordered mesoporous silica film/indium tin oxide electrode

**FIGURE 1:**
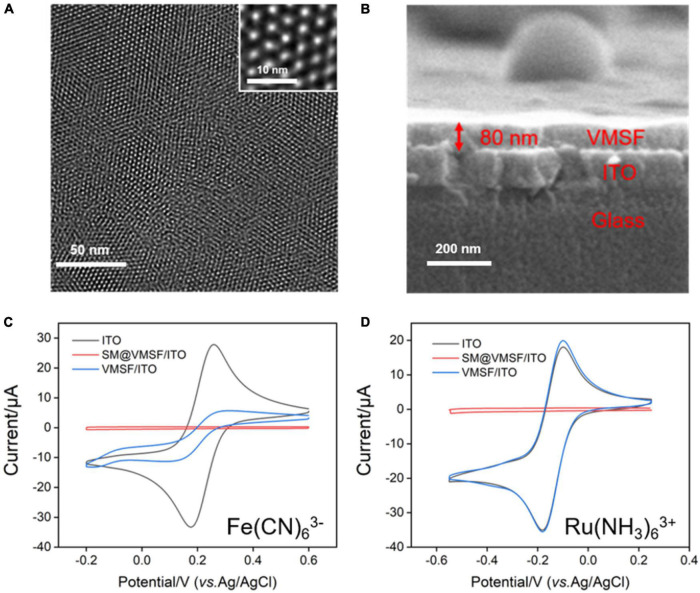
**(A)** Top-view TEM and **(B)** cross-sectional view SEM images of VMSF. The inset in **(A)** is the corresponding magnified image. CV curves obtained at the bare ITO, SM@VMSF/ITO, and VMSF/ITO electrodes in 0.05 M KHP containing 0.5 mM K_3_Fe(CN)_6_ **(C)** or Ru(NH_3_)_6_Cl_3_ **(D)**. The scan rate was 50 mV/s.

The morphology and thickness of VMSF were characterized using TEM and SEM. Figures 1A,B shows top-view TEM (a) and cross-sectional view SEM and (b) images of VMSF. Top-view TEM image shows that the pores of VMSF are highly ordered with uniform pore size and intact over large area. From the enlarged view of VMSF displayed in the inset of Figure 1A, nanopores are regularly aligned in hexagonal shapes and have a diameter of ca. 2.4 nm. The cross-sectional view SEM image shows that the as-prepared VMSF/ITO electrode is divided into three layers, namely VMSF layer, ITO layer, and glass substrate (Figure 1B). Moreover, the thickness of VMSF is rather uniform, and its thickness is measured to be about 80 nm. Supplementary Figure 1 shows the top-view TEM image of SM@VMSF. As compared with that of VMSF (Figure 1A), the removal of SM from the nanochannels could not cause any significant changes in morphology, indicating the stability of the VMSF on the ITO surface. The electrochemical method was then employed to examine the integrity and charge permselectivity of VMSF. Two commonly used electrochemical probes with opposite charges [Fe(CN)_6_^3–^ and Ru(NH_3_)_6_^3+^] were used. Figures 1C,D compares the cyclic voltammetry (CV) curves of Fe(CN)_6_^3–^ and Ru(NH_3_)_6_^3+^ at the bare ITO, SM@VMSF/ITO, and VMSF/ITO electrodes. As presented, both Fe(CN)_6_^3–^ and Ru(NH_3_)_6_^3+^ are not able to generate Faradaic currents at the SM@VMSF/ITO electrode (red line), which is because of the hindrance effect of micelles inside the silica nanochannels and further proves that the as-prepared VMSF is intact and crack-free. The presence of electrochemical current signals for these two probes at the VMSF/ITO electrode (blue line) suggests that the silica nanochannels are open and allow the molecular transport from the solution to the underlying electrode surface. In addition, the VMSF/ITO electrode exhibits apparent charge permselectivity compared with the bare ITO electrode, namely electrostatically attracting the access of positively charged Ru(NH_3_)_6_^3+^ probe and suppressing that of Fe(CN)_6_^3–^ probe. This phenomenon was due to the negatively charged channel walls of VMSF in the experimental conditions (p*K*_*a*_ of silanol groups ∼2). Above results indicate the preparation of VMSF/ITO electrode is successful.

### Electrochemical behavior of Ru(bpy)_3_^2+^ at the vertically ordered mesoporous silica film/indium tin oxide electrode

**FIGURE 2:**
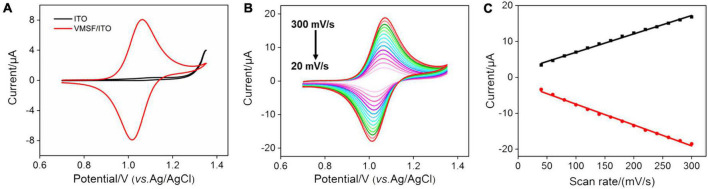
**(A)** CV curves obtained at the bare ITO (black line) and VMSF/ITO (red line) electrodes in 0.1 M PBS (pH 7.0) containing 1 μM Ru(bpy)_3_^2+^. The scan rate was 100 mV/s. **(B)** CV curves of the VMSF/ITO electrode in 0.1 M PBS (pH 7.0) containing 1 μM Ru(bpy)_3_^2+^ at different scan rates. **(C)** The relationship between redox peak currents and scan rate.

Due to the electrochemical response of G which depends on the redox signal of Ru(bpy)_3_^2+^, it is necessary to study the electrochemical behavior of Ru(bpy)_3_^2+^ at the VMSF/ITO electrode. To verify the accumulation ability of VMSF toward Ru(bpy)_3_^2+^, we compared the CV curves obtained at the ITO and VMSF/ITO electrodes in 0.1 M PBS (pH 7.0) containing 1 μM Ru(bpy)_3_^2+^ (Figure 2A). Arising the high overpotential of G at the bare ITO electrode, no obvious redox peaks are observed. It can be seen that the VMSF/ITO electrode has a significantly enhanced electrochemical response toward Ru(bpy)_3_^2+^, which is attributed to the electrostatic attraction effect between positively charged Ru(bpy)_3_^2+^ and negatively charged channel walls of VMSF, and the magnitude of the oxidation peak current measured by VMSF/ITO electrode was 8.20 μA, which was about 20-fold larger than that of ITO electrode (0.42 μA). The CV curves of the VMSF/ITO electrode in 0.1 M PBS (pH 7.0) containing 1 μM Ru(bpy)_3_^2+^ at different scan rates were recorded (Figure 2B). As displayed, the values of redox peak currents increased with increasing scan rate, yielding a linear relationship in the range of 20–300 mV/s (Figure 2C). This indicates the electrochemical reaction of Ru(bpy)_3_^2+^ on the VMSF/ITO electrode surface is adsorption-controlled.

### Optimal conditions for Ru(bpy)_3_^2+^-mediated electrochemical sensor

Two types of electrodes (bare ITO and VMSF/ITO) and four types of buffer solutions [PBS, NaAc-HAc, Britton-Robinson buffer (BRB), and Tris–HCl] were first employed to obtain optimal detection performance. As shown in Supplementary Figure 2, compared with bare ITO, VMSF/ITO is more sensitive for G determination, due to the electrostatic enrichment effect of Ru(bpy)_3_^2+^ by VMSF. Thus, VMSF/ITO electrode was chosen as the electrode for the determination of G. There is no obvious change for these four supporting electrolytes (Supplementary Figure 3). A typical buffer solution, PBS, was chosen as the electrolyte solution for the determination of G.

**FIGURE 3:**
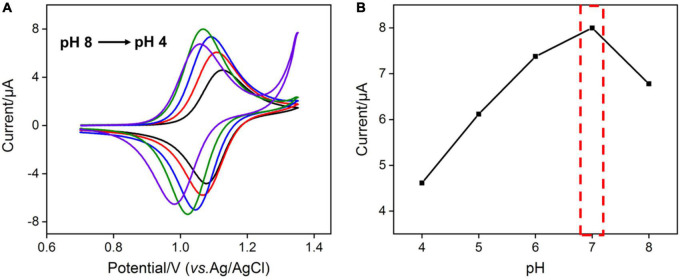
**(A)** CV curves obtained at VMSF/ITO electrode in 0.1 M PBS with different pH containing 1 μM Ru(bpy)_3_^2+^. The scan rate was 100 mV/s. **(B)** The relationship between anodic peak currents and pH.

**FIGURE 4:**
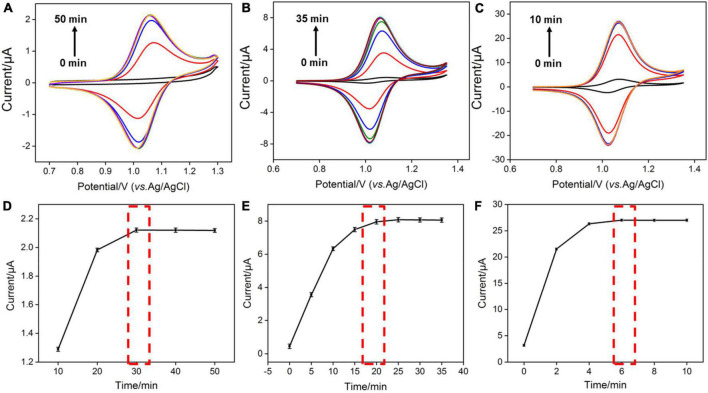
**(A–C)** CV curves obtained at the VMSF/ITO electrode with different accumulation time in 0.1 M PBS solution (pH 7.0) containing various concentrations of Ru(bpy)_3_^2+^. The scan rate was 100 mV/s. **(D–F)** The relationship between anodic peak currents and accumulation time.

As the amount of redox mediator Ru(bpy)_3_^2+^ inside the silica nanochannels is important for the electrochemical response of G, the pH of the supporting electrolyte and concentration of Ru(bpy)_3_^2+^ used in solution were also optimized to realize a highly sensitive performance for the detection of G. As shown in Figure 3A, with the pH value of the supporting electrolyte increases, the redox signals of Ru(bpy)_3_^2+^ increase and reach the highest at the pH value of 7.0. This phenomenon is attributed to the stronger negative surface of VMSF at a higher pH. However, the redox peak currents of Ru(bpy)_3_^2+^ decrease with further increasing the pH (8.0) (Figure 3B), which is probably due to the instability of the VMSF under alkaline conditions, and the effect of the pH of the supporting electrolyte on the G detection performance is further studied. As shown in Supplementary Figure 4, when the pH increases from 4 to 7, the obtained anodic current signal enhances. Therefore, pH = 7 was selected for the following experiments. Three kinds of Ru(bpy)_3_^2+^ concentrations were employed, namely 0.1, 1, and 10 μM (Figure 4). It could be found that 30, 20, and 6 min were the corresponding optimal accumulation time for Ru(bpy)_3_^2+^ at the VMSF/ITO electrode, respectively. In addition, more accumulation time is required for lower concentration of Ru(bpy)_3_^2+^, and the obtained anodic peak current is lower, which is due to the slower diffusion of Ru(bpy)_3_^2+^ from bulk solution to the underlying ITO electrode surface through the silica nanochannels and less amount of Ru(bpy)_3_^2+^ on the electrode surface.

### Electrochemical detection of guanine using vertically ordered mesoporous silica film/indium tin oxide electrode and Ru(bpy)_3_^2+^ mediator

**FIGURE 5:**
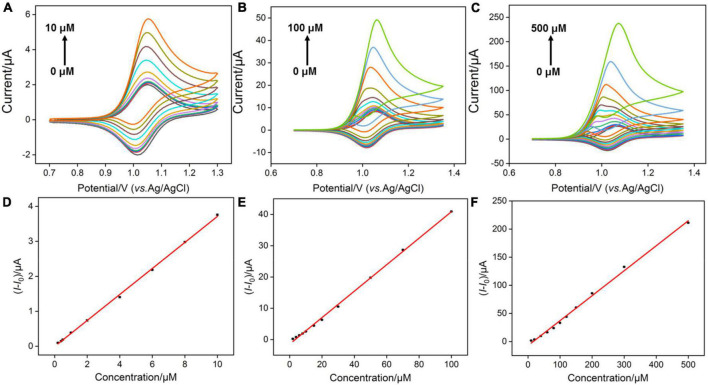
**(A–C)** CV curves obtained at the VMSF/ITO electrode with successive addition of different concentrations of G after accumulating different concentrations of Ru(bpy)_3_^2+^. The scan rate was 100 mV/s. **(D–F)** The linear relationship between the variation of anodic peak currents (*I*-*I*_0_) and concentrations of G. *I* and *I*_0_ denote the anodic peak currents in the presence and absence of G, respectively.

According to the previous reports (16), Ru(bpy)_3_^3+^ oxidized from Ru(bpy)_3_^2+^ could mediate the electrochemical oxidation of G, displaying electrocatalytic ability toward G oxidation and leading to the enhanced anodic peak current in CV curves. Under optimal buffer pH and preconcentration time of Ru(bpy)_3_^2+^, we test the CV responses of the VMSF/ITO electrodes toward various concentrations of G in 0.1 M PBS (pH 7.0) containing 0.1 μM, 1 M m, and 10 μM Ru(bpy)_3_^2+^, respectively. As shown in Figures 5A–C, with increasing the G concentration, enhanced anodic peak currents and decreased cationic peak currents are observed for all cases, showing a typical characteristic of electrocatalytic oxidation. Electrocatalytic currents exhibit a good relationship with the concentration of G (Figures 5D–F), and the analytical performances are summarized in Table 1. When 0.1 μM Ru(bpy)_3_^2+^ is present in the bulk solution, the VMSF/ITO electrode is able to detect G in the range from 0.2 to 10 μM with a detection of limit (LOD) of 58 nM and the achieved linear regressive equation is I (μA) = 0.372C (μM)–0.00588 (*R*^2^ = 0.999). Moreover, analytical performances of the proposed sensor toward G vary with the amount of Ru(bpy)_3_^2+^ mediator. A higher lowest detection concentration and LOD were obtained at the high concentration of Ru(bpy)_3_^2+^, showing the adjustability of our sensing strategy. The detection performances of this method for G in terms of linear range and LOD were also compared with other reported electrochemical sensors, as shown in Table 2. As revealed, such simple VMSF/ITO sensor integrated with Ru(bpy)_3_^2+^ mediator has a wide linear range and a relatively low LOD, which shows great potential for practical applications.

**TABLE 1:** Comparison of G detection performance with different mediator contents.

Ru(bpy)_3_^2+^ concentration (μM)	Accumulation time (min)	Linear range (μM)	LOD (μM)	Sensitivity (μA/μM)
0.1	30	0.2–10	0.0580	0.372
1	20	2–100	0.242	0.423
10	6	10–500	1.60	0.444

**TABLE 2:** Comparison of the electrochemical performances of VMSF/ITO for G detection with other reported electrochemical sensors.

Electrodes	Techniques	Linear range (μM)	LOD (μM)	References
MWCNT-Fe_3_O_4_@PDA-Ag/CPE	DPV	8–130	1.47	(11)
Fe_2_V_4_O_13_ NPs/CPE	DPV	0.5–60	0.032	(13)
ZnS NPs/CPE	DPV	1–15	0.038	(12)
PTCA-MWCNTs/GCE	LSV	0.759–20.9	0.0253	(47)
NiFe PBA HNCs/Nafion/GCE	i-t	50–1400	0.0104	(48)
VMSF/rGO/GCE	DPV	0.2–200	0.096	(49)
VMSF/ITO	CV	0.2–500	0.058	This work

MWCNT, multi-walled carbon nanotube; PDA, polydopamine; CPE, carbon paste electrode; NPs, nanoparticles; PTCA, 3,4,9,10-perylene tetracarboxylic acid; GCE, glassy carbon electrode; PBA HNCs, prussian blue analogs hollow nanocubes; rGO, reduced graphene oxide.

### Anti-interference ability of the Ru(bpy)_3_^2+^-mediated vertically ordered mesoporous silica film/indium tin oxide sensor

**FIGURE 6:**
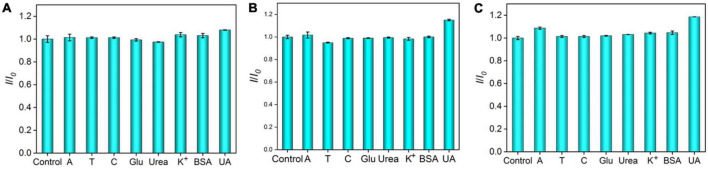
Anodic peak current ratio (*I*/*I*_0_) at the VMSF/ITO electrode in PBS (0.1 M, pH 7.0) containing 0.1 μM Ru(bpy)_3_^2+^ and 0.5 μM G **(A)**, 1 μM Ru(bpy)_3_^2+^ and 5 μM G **(B)**, 10 μM Ru(bpy)_3_^2+^, and 10 μM G **(C)** in the absence (*I*_0_) or presence (*I*) of the interferents. The concentration of interfering species was 10-fold higher than G.

Anti-interference ability is an important characteristic of electrochemical sensor. DNA has four important bases, namely, adenine (A), cytosine (C) thymine (T), and G, and most biological samples, such as plasma, urea, uric acid (UA), and glucose (Glu), are often presented Therefore, interfering substances (A, T, C, Glu, urea, K^+^, UA, and BSA) with 10-time higher concentrations than G are chosen to evaluate the anti-interference capacity of the Ru(bpy)_3_^2+^-mediated VMSF/ITO sensor. As shown in Figure 6, there is no obvious effect in detecting G at the low concentrations of Ru(bpy)_3_^2+^. However, under medium or high concentration of Ru(bpy)_3_^2+^, other substances except UA have a slight effect on the detection of G. This is because UA as a reducing agent could affect the electrocatalytic oxidation of Ru(bpy)_3_^2+^ by G.

### Detection of guanine in beer samples

**FIGURE 7:**
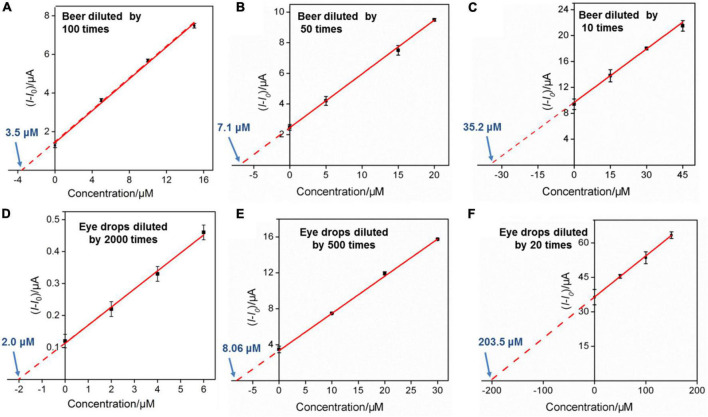
**(A–C)** Linear relationship between the anodic peak current variation and G concentration in beer sample. The detected solution contains 1 μM Ru(bpy)_3_^2+^. **(C,D)** The linear relationship between the anodic peak current variation and ganciclovir concentration in ganciclovir eye drops sample. The detected solution contains 0.1 μM Ru(bpy)_3_^2+^ **(D)**, 1.0 μM Ru(bpy)_3_^2+^ **(E)**, and 10 μM Ru(bpy)_3_^2+^ **(F)**, respectively.

Studies show that the content of G in common beer sold on the market is generally high. Uric acid is the final metabolite of G in the human body, and excessive content will easily cause gout. Therefore, accurate and convenient determination of G in beer is highly desirable for human health. Beer samples diluted by 100, 50, and 10 times using 0.1 M PBS (pH 7.0) were used directly for real sample analysis, and the results are shown in Figures 7A–C. The obtained original G contents in beer samples are 350 μM, 355 M m, and 349 μM, respectively, by using standard addition method, which are similar to the labeled value of beer (350 μM) and the results detected by HPLC (359 μM), proving the reliability of our method. Besides, by comparing the sensitivity of G detection in buffer solution and beer samples, matrix effect is indeed existed in beer samples. However, arising from the anti-fouling capacity of VMSF, the quantitative determination of G could not be affected. Moreover, direct analyses of G in spinach and apple samples were studied, and the results are shown in Supplementary Table 1, further demonstrating the potential of our sensor for direct and reliable detection of G in complex real samples.

### Detection of ganciclovir in eye drops

**FIGURE 8:**
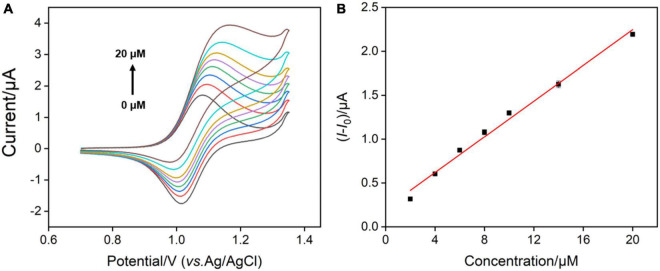
**(A)** CV curves measured by VMSF/ITO after adding different concentrations of ganciclovir to the 0.1 M PBS (pH 7.0) containing 0.1 μM Ru(bpy)_3_^2+^. **(B)** The linear relationship between anodic peak current variation and concentration of ganciclovir.

To explore the universality of the proposed sensor, G analog with similar structure (e.g., ganciclovir (2-amino-9-((1,3-dihydroxypropan-2-yloxy)methyl)-1*H*-purin-6-one)) was also determined. Ganciclovir as a common antiviral drug is often used to prevent or treat cytomegalovirus disease in transplant patients (46). Figure 8A shows the CV curves of the VMSF/ITO electrode in 0.1 M PBS (pH 7.0) containing 0.1 μM Ru(bpy)_3_^2+^ and various concentrations of ganciclovir. Similar electrocatalytic oxidation was displayed, and a linear range from 2 to 20 μM was obtained (Figure 8B). Moreover, the content of ganciclovir in commercially available ganciclovir eye drops was detected. The detected eye drop samples were obtained by adding 5, 20, and 500 μL ganciclovir eye drops to 10 mL 0.1 M PBS (pH 7.0) containing 0.1 μM Ru(bpy)_3_^2+^, 1 μM Ru(bpy)_3_^2+^, and 10 μM Ru(bpy)_3_^2+^, respectively. After the successive addition of various concentrations of standard ganciclovir to the above eye drop sample, electrocatalytic signals were recorded and the results are shown in Figures 7D–F. As seen, the detected concentrations of ganciclovir (4.00, 4.03, and 4.07 mM) in eye drops for various diluted times (2000, 500, and 20 times) were very close to the theoretical value (3.90 mM) shown on the medicine instruction, which also were very close to the results determined by HPLC (3.95 mM). Note that a low amount of expensive Ru(bpy)_3_^2+^ (0.1 μM) and ultrasmall sample volume (5 μL) used here make our sensor suitable for practical analytical applications.

## Conclusion

In summary, we have demonstrated an electrochemical method for highly sensitive detection of G by the combination of Ru(bpy)_3_^2+^-mediated electrocatalytic oxidation and VMSF/ITO electrode-assisted amplification strategy. VMSF was simply prepared on the ITO electrode by EASA method in a few seconds without the use of adhesive layer or pretreatment of the electrode and able to serve as enrichment material for the signal amplification of Ru(bpy)_3_^2+^ mediator. Thus, Ru(bpy)_3_^2+^ inside the nanochannels of VMSF acting as an electron shuttle shows electrocatalytic oxidation toward G, producing the increased anodic peak current and enabling the quantitative determination of G. Arising from the electrostatic effect of VMSF and mediated capacity of Ru(bpy)_3_^2+^, electrochemical detection performance for G is related to the pH of the supporting electrolyte and the content of Ru(bpy)_3_^2+^. Owing to the good anti-fouling and anti-interference ability of VMSF, this Ru(bpy)_3_^2+^-mediated electrochemical strategy has shown satisfactory results in direct analysis of G or G analog in beer and ganciclovir eye drops, which could be applied to other real samples (e.g., spinach) by simply adjusting the used content of Ru(bpy)_3_^2+^. The proposed sensing strategy could extend the analytical application of VMSF-based sensors and hold the potential of designing DNA-based sensors for a wide range of analytes. However, the determination of G could be influenced when other reducing substances (uric acid) coexist in the samples.

## Data availability statement

The original contributions presented in this study are included in the article/Supplementary material, further inquiries can be directed to the corresponding authors.

## Author contributions

LY, TZ, and HZ curated the data. FY handled the supervision, reviewing, editing, and writing of the manuscript. YL wrote and edited the manuscript. All authors contributed to the article and approved the submitted version.
