# Supplementary material for: Silica nanochannels boosting Ru(bpy)32+-mediated electrochemical sensor for the detection of guanine in beer and pharmaceutical samples
Source: Front Nutr. 2022 Aug 30;9:987442. doi: 10.3389/fnut.2022.987442 (PMC9468770; doi:10.3389/fnut.2022.987442)
Supplement: Supplementary file 1 [file Data_Sheet_1.pdf]

Silica nanochannels boosting $Ru(bpy)_3^{2+}$-mediated electrochemical sensor for detection of guanine in beer and pharmaceutical samples

Luoxing Yang[a‡], Tongtong Zhang[b‡], Huaxu Zhou[a], Fei Yan[a*] and Yan Liu[c*]

[a] Key Laboratory of Surface & Interface Science of Polymer Materials of Zhejiang Province, Department of Chemistry, Zhejiang Sci-Tech University, Hangzhou, China.

[b] Department of Hepatobiliary and Pancreatic Surgery, The Center for Integrated Oncology and Precision Medicine, Affiliated Hangzhou First People's Hospital, Zhejiang University School of Medicine, Hangzhou, China

[c] Guangxi Medical University Cancer Hospital, Nanning, China.

*** Correspondence:**

Corresponding authors: feifei19881203@126.com; liuyan@gxmu.edu.cn

Fei Yan: 0000-0002-2822-698X

Table of Contents

S1 TEM characterization of SM@VMSF

S2 Optimization of experimental conditions

S2.1 Types of electrodes

S2.2 Types of buffer solution

S2.3 pH of supporting electrolyte

S3 Electrochemical detection of G in spinach and apple samples

S1 TEM characterization of SM@VMSF

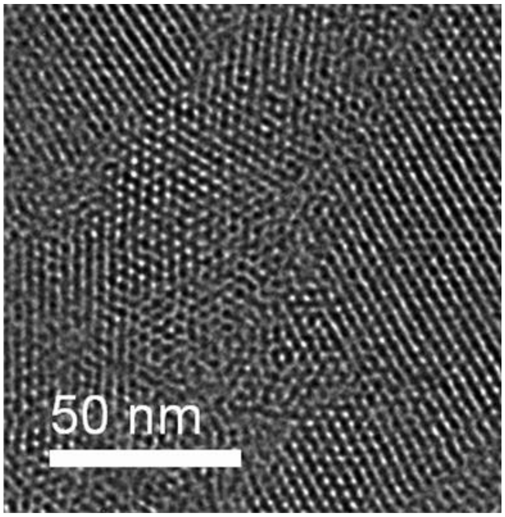

Fig. S1 Top-view TEM image of SM@VMSF.

S2 Optimization of experimental conditions

S2.1 Types of electrodes

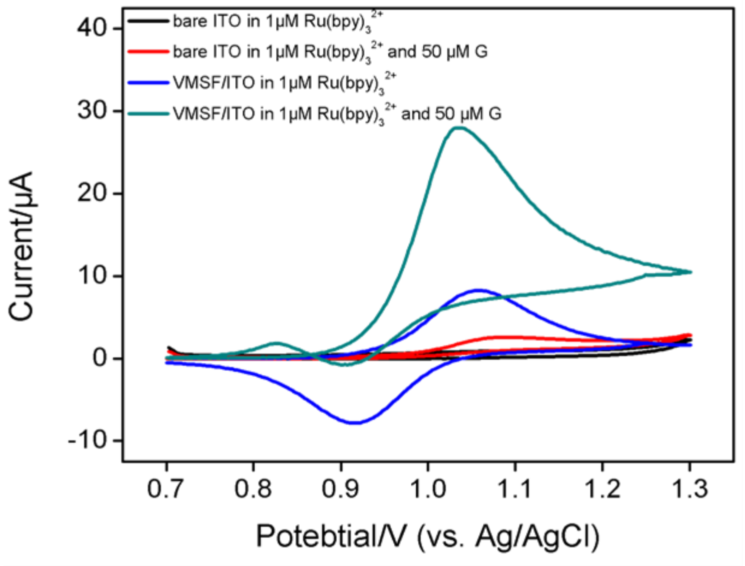

Fig. S2 CV curves of bare ITO and VMSF/ITO electrodes in 0.1 M PBS (pH = 7.0) containing 1 μM $Ru(bpy)_3^{2+}$ with or without 50 μM G.

S2.2 Types of buffer solution

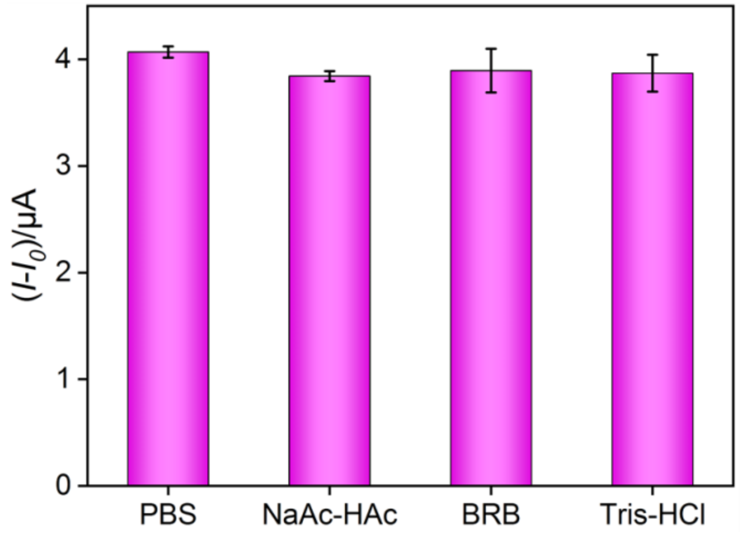

Fig. S3 Effect of different buffer solution (0.1 M, pH = 7.0) containing 1 μM $Ru(bpy)_3^{2+}$ on the detection of G (10 μM) at the VMSF/ITO electrode.

S2.3 pH of supporting electrolyte

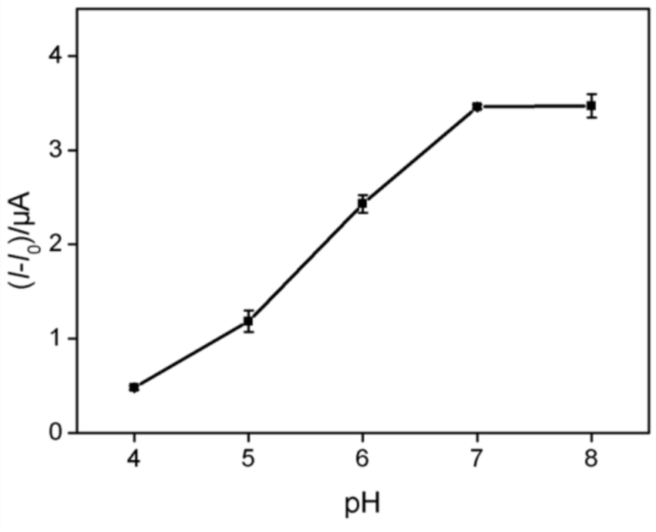

Fig. S4 The relationship between the variation of anodic peak currents (I-I_0) and pH. I and I_0 denote the anodic peak currents in the presence and absence of G, respectively. The detected solution is 0.1 M PBS with different pH containing 1 μM $Ru(bpy)_3^{2+}$ and the concentration of G is 8 μM.

S3 Electrochemical detection of G in spinach and apple samples

Table S1 Electrochemical detection of G in spinach and apple samples (n = 3)

Real samples [a]	Added (μM)	Found by HPLC (μM)	Found by our method (μM)	Recovery [b] (%)	RSD (%)
Spinach	0.00	11.1	10.8	/	0.5
	10.0	22.1	21.4	106	0.7
	20.0	30.0	30.7	99.5	2.5
Apple	0.00	/	/	/	/
	5.00	5.10	5.02	100	0.4
	10.0	9.90	9.74	97.4	0.5

[a] spinach and apple are diluted 100 times and 10 times, respectively.

[b] Recovery = (determined C_G of spiked sample − determined C_G of non-spiked sample)/added value × 100%
